# The Role of Arginase 1 in Post-Stroke Immunosuppression and Ischemic Stroke Severity

**DOI:** 10.1007/s12975-015-0431-9

**Published:** 2015-10-30

**Authors:** Ashley B. Petrone, Grant C. O’Connell, Michael D. Regier, Paul D. Chantler, James W. Simpkins, Taura L. Barr

**Affiliations:** Department of Neurobiology and Anatomy, West Virginia University, One Medical Center Drive, Morgantown, WV 26508 USA; Center for Basic and Translational Stroke Research, West Virginia University, One Medical Center Drive, Erma Byrd 119, Morgantown, WV 26508 USA; Department of Pharmaceutical Sciences, West Virginia University, One Medical Center Drive, Morgantown, WV 26508 USA; Department of Biostatistics, West Virginia University, One Medical Center Drive, Morgantown, WV 26508 USA; Department of Exercise Physiology, West Virginia University, One Medical Center Drive, Morgantown, WV 26508 USA; Center for Cardiovascular and Respiratory Sciences, West Virginia University, One Medical Center Drive, Morgantown, WV 26508 USA; Department of Physiology and Pharmacology, West Virginia University, One Medical Center Drive, Morgantown, WV 26508 USA; School of Nursing, West Virginia University, One Medical Center Drive, Morgantown, WV 26508 USA

**Keywords:** Stroke, Immune system, Arginase, Neutrophil-lymphocyte ratio

## Abstract

A balanced immune system response plays an important role in acute ischemic stroke (AIS) recovery. Our laboratory has previously identified several immune-related genes, including arginase 1 (*ARG1*), with altered expression in human AIS patients. The neutrophil-lymphocyte ratio (NLR) may be a marker of the degree of immune dysregulation following AIS; however, the molecular mechanisms that may mediate the NLR are unknown. The purpose of this study was to (1) examine the relationship between ARG1, NLR, and AIS severity and (2) to utilize principal component analysis (PCA) to statistically model multiple gene expression changes following AIS. AIS patients and stroke-free control subjects were recruited, and blood samples were collected from AIS patients within 24 h of stroke symptom onset. White blood cell differentials were obtained at this time to calculate the NLR. Gene expression was measured using real-time PCR. PCA with varimax rotation was used to develop composite variables consisting of a five-gene profile. *ARG1* was positively correlated with NLR (*r* = 0.57, *p* = 0.003), neutrophil count (*r* = 0.526, *p* = 0.007), NIHSS (*r* = 0.607, *p* = 0.001), and infarct volume (*r* = 0.27, *p* = 0.051). PCA identified three principal components that explain 84.4 % of variation in the original patient gene dataset comprised of *ARG1*, *LY96*, *MMP9*, *s100a12*, and PC1 was a significant explanatory variable for NIHSS (*p* < 0.001) and NLR (*p* = 0.005). Our study suggests a novel relationship between ARG1, NLR, and stroke severity, and the NLR is an underutilized clinically available biomarker to monitor the post-stroke immune response.

## Introduction

A balanced immune system response plays an important role in acute ischemic stroke (AIS) recovery [1, 2]. AIS results in a well-described immediate, but non-specific innate inflammatory response. Innate inflammatory processes activate adaptive immune cells responsible for controlling inflammation, modulating ischemic damage, and promoting neurogenesis [3]. In some patients, for reasons poorly understood, the adaptive immune system is incapable of responding to the innate inflammatory signals, resulting in post-stroke immune suppression. Stroke-induced adaptive immune suppression is associated with greater stroke severity, increased risk of infection, and poor recovery, and is characterized by decreased T lymphocyte function and peripheral cell counts and increased inflammatory cytokines [1, 4]. Human stroke genomic biomarker studies have identified a common panel of five genes responding to AIS in the peripheral blood: arginase 1 (*ARG1*), lymphocyte antigen 96 (*LY96*), matrix metalloproteinase 9 (*MMP9*), s100 calcium-binding protein A12 (*s100A12*), and chemokine CC motif receptor 7 (*CCR7*) [5, 6]. Our laboratory has also shown that the neutrophil-lymphocyte ratio (NLR) is a simple, inexpensive biomarker that may be used to predict outcome following AIS and may be a clinically available marker of the degree of immune dysregulation following AIS [7, 8]; however, the relationship between the NLR and these genomic biomarkers following AIS is unknown.

*ARG1* is consistently upregulated in the whole blood of AIS patients [5, 6]. The traditional role for the enzyme ARG1 is catalyzing the metabolism of the substrate l-arginine to l-ornithine and urea [9]. Because ARG1 and nitric oxide synthase (NOS) share l-arginine as a common substrate, ARG1 can be viewed as a competitive inhibitor of NOS. In addition to this traditional role, ARG1, derived from innate immune cells, has been shown to mediate the adaptive immune response [10]. In humans, *ARG1* expression is highest in neutrophils compared to other peripheral blood leukocytes [5], and ARG1 protein released from neutrophils suppresses T lymphocyte proliferation through downregulation of T lymphocyte CD3ζ chain [10]. Until very recently, ARG1-induced lymphocyte suppression had not been shown in the context of AIS. Sippel et al. reported that ARG1 protein released from neutrophils induces lymphopenia in a murine model of stroke [11]. Given the differences between murine and human immune systems [12], it remains to be determined whether ARG1 induces immunosuppression in human AIS patients.

The purpose of this pilot study was to (1) examine the relationship between ARG1, NLR, and AIS severity; we hypothesized that increased *ARG1* expression and serum protein activity is associated with an increased NLR, giving rise to increased AIS severity and poor outcome; (2) to validate the role of ARG1 as a novel biomarker of immune suppression in AIS; and (3) to utilize principal component analysis (PCA) to statistically model multiple gene expression changes following AIS.

## Materials and Methods

### Subject Recruitment

Informed consent was obtained from all individual participants included in the study. AIS patients and stroke-free control subjects were recruited from Ruby Memorial Hospital (Morgantown, WV). Male and female AIS patients were eligible for recruitment if the following inclusion criteria were met: (1) age ≥18 years, (2) confirmation of acute stroke by neuroimaging (CT or MRI), and (3) had blood drawn within 24 h of symptom onset/“last-known normal,” prior to thrombolysis or interventional treatment. Patient data from the medical record were reviewed and recorded, including (1) National Institutes of Health Stroke Scale (NIHSS); (2) clinical laboratory analyses, including white blood cell differential; and (3) brain imaging (CT/ MRI). Stroke-free control subjects were eligible for recruitment if the following inclusion criteria were met: (1) age ≥18 years; (2) no history of AIS, transient ischemic attack, brain injury, or other overt central nervous system disease; and (3) recent hospitalization. Medical histories were obtained directly from stroke-free control subjects; however, complete access to medical records of control subjects was not available.

### Infarct Volume Calculation

Brainlab iPlan® software was used to manually trace and calculate infarct volume from either CT or MRI images. All images were obtained within 24 h of symptom onset, regardless of modality. All infarct volume calculations were verified by a neuroradiologist at Ruby Memorial Hospital (Morgantown, WV).

### Research Protocol Approval and Informed Consent

This study received approval for human subject research from the institutional review boards of West Virginia University and Ruby Memorial Hospital (Morgantown, WV). Written informed consent was obtained from all subjects or their authorized representatives prior to performing study procedures.

### Blood Collection

Peripheral venous whole blood was drawn from stroke subjects not later than 24 h of stroke symptom onset. Blood was collected into PAXgene® Blood RNA tubes (Becton-Dickinson). Immediately after blood collection, tubes were inverted 8–10 times and stored at −80 °C until analysis. Whole blood was collected in serum separator tubes, centrifuged at 4000 g, aliquoted into microcentrifuge tubes within 1 h of collection, and stored at −80 °C until analysis.

### RNA Extraction

PAXgene® Blood RNA tubes were thawed overnight (16–20 h) at room temperature prior to RNA extraction. The PAXgene Blood RNA kit (Pre-Analytix) was used to purify/extract intracellular RNA, per manufacturer’s instructions. RNA concentration and quality was determined by absorbance using a Take3 Trio Microplate (BioTek®) read on a Syntek Hybrid Plate Reader and analyzed using Gen5 (BioTek) software. A260/A280 values between 1.8 and 2.2 were considered acceptable RNA quality.

### Gene Expression Analysis

RNA was converted to complementary DNA (cDNA) using the High-Capacity Reverse Transcription Kit (Applied Biosystems). cDNA (10 ng) was used for quantitative real-time principal component regression (PCR) amplification using SYBR Green chemistry using the Rotor-Gene Q real-time PCR cycler (Qiagen). The following Quantitect primers (Qiagen) were used: ARG1 (NM_000045, NM_001244438), LY96 (NM_015364), MMP9 (NM_004994), s100a12 (NM_005621), and CCR7 (NM_001838). Gene expression was normalized using both PPIB (NM_000942) and B2M (NM_004048). These reference genes are known to be stably expressed in whole blood of ischemic stroke patients [13]. We confirmed that both PPIB and B2M were stably expressed across our subject population (SD < 1) (unpublished data). Fold change differences were calculated by the ΔΔCT method [14].

### Measurement of Serum ARG1 Protein Activity

Serum ARG1 protein activity levels were determined in AIS patients using an Arginase Activity Assay Kit (Sigma-Aldrich). All procedures were performed per manufacturer’s instructions. Activity data is presented in units per liter.

### Sample Size Estimation

Post hoc power analyses were conducted to determine achieved power, given alpha level, sample size, and effect size using G*Power3 [15]. We performed two independent power analyses for the two primary aims of the study: (1) to evaluate the relationship of ARG1 and stroke severity, as measured by NIHSS, and (2) to compare ARG1 expression between stroke and control. Using a linear regression model, controlling for seven predictors (age, sex, smoking, hypertension, hyperlipidemia, diabetes, and prior stroke), we determined that our sample size of *n* = 26 and effect size 0.5 was sufficient to detect differences in ARG1 expression along the NIH stroke severity score scale with 88 % power at an alpha level of 0.05. Using a linear regression model, controlling for eight predictors (case (control v. stroke), age, sex, smoking, hypertension, hyperlipidemia, diabetes, and prior stroke), we determined that our sample size *n* = 45 and effect size 0.5 was sufficient to detect differences in ARG1 expression between stroke and controls with 99 % power at an alpha level of 0.05.

### Statistical Analysis

The Mann–Whitney *U* test was used for testing differences between cases and controls for continuous variables. The Fisher’s exact test was used to compare cases and control for categorical variables. Spearman’s correlation, using the transformed variables, with a continuity correction was used for continuous variable bivariate relationships.

If the variable’s skew was greater than 0.5, the variable was transformed using the Box-Cox transformation. If there were negative values, we used the exponential transformation. The application of the Box-Cox and exponential transformations provided substantial reductions in variable skew and hence mitigated the presence of variable specific outliers. All variables were scaled by their standard deviation prior to applying any transformation.

PCA with varimax rotation was used to develop composite variables consisting of a five-gene profile. The relevant components were chosen such that at least 80 % of the original variation in the data was explained. Following the identification of the principal components, principal component regression was performed to assess the relationship between the derived components and the outcomes of interest.

Linear regression model selection used Akaike information criterion (AIC) backwards, stepwise selection to identify informative variables from among the set of candidate regressors (age, sex, smoking, hypertension, hyperlipidemia, diabetes, and prior stroke). All statistical analyses were performed using the R software environment for statistical computing and graphics. Statistical significance was taken at the 5 % alpha level.

## Results

### Clinical Characteristics

Forty-five subjects (26 ischemic stroke patients and 19 control subjects) were recruited for this study. All stroke patients were confirmed definite ischemic stroke by neuroimaging (CT or MRI) [16]. There was no difference in gender between the groups; however, stroke patients were significantly older (*p* < 0.001). Hypertension (*p* = 0.007) and smoking (*p* = 0.014) were more common in the stroke group compared to controls. The mean NIHSS score of the stroke patients at baseline (<24 h after symptom onset) was 8.65 (range 0–28, SD = 7.26). Of the 26 strokes, 11 were mild (42 %), 6 were moderate (23 %), and 9 were scored as severe (35 %). Ten stroke patients (38 %) received rTPA; however, all patients had blood drawn prior to rTPA administration or other intervention. The mean time from symptom onset/last-known normal was 7 h and 30 min (Table 1).

**Table 1:** Univariate associations between stroke and control subjects

	Total sample (*n* = 45)	Stroke (*n* = 26)	Control (*n* = 19)	Statistic/*df*	*p* value
Age, years, mean ± SD	63.2 ± 16.5	70.9 ± 16.1	52.7 ± 10.3	*T*–4.334/43	.000**
Sex, *n* (% female)	30 (67)	15 (58)	15 (79)	*X* ^2^–2.232/1	.135
NIH stroke scale score, mean ± SD	8.65 ± 7.25	8.65 ± 7.25	0	*T*–5.183/1	.000**
Received tPA (%)	10 (22)	10 (38)	0	*X* ^2^–9.396/1	.002**
Previous ischemic stroke (%)	8 (17)	7 (27)	1 (7)	*X* ^2^–3.523/1	.061
Hypertension (%)	25 (56)	21 (80)	4 (27)	*X* ^2^–7.162/1	.007**
Diabetes (%)	8 (18)	6 (23)	2 (13)	*X* ^2^–1.183/1	.277
Dyslipidemia (%)	18 (40)	13 (50)	5 (33)	*X* ^2^–0.770/1	.380
Current smoker (%)	7 (16)	7 (27)	0	*X* ^2^–6.058/1	.014**

**Significant differences between stroke and control (*p* ≤ 0.05)

### ARG1 mRNA Expression, NLR, and Stroke Severity

**Fig. 1:**
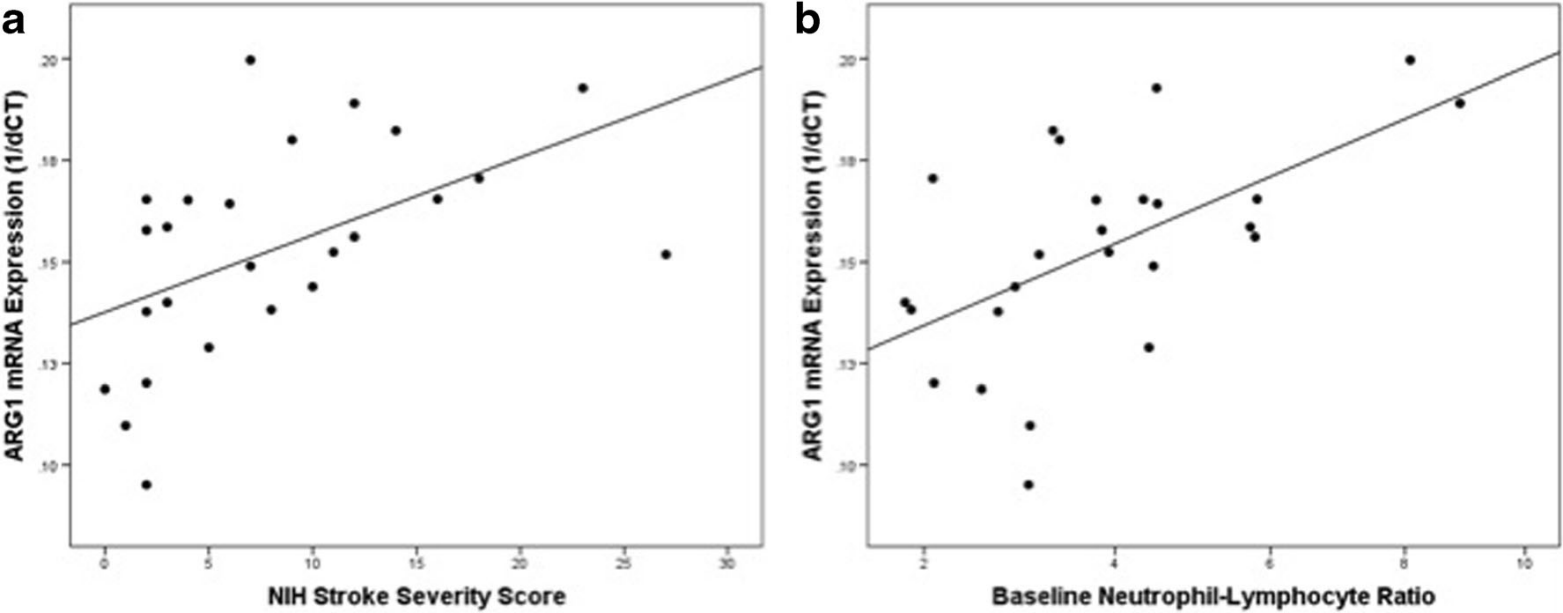
The relationship between *ARG1* mRNA expression, NIHSS, and NLR. **a** *ARG1* expression is positively correlated with NIHSS (*r* = 0.607, *p* < 0.001). **b** *ARG1* expression is positively correlated with NLR (*r* = 0.582, *p* = 0.002)

Stroke severity was analyzed as both NIHSS and infarct volume. *ARG1* was positively correlated with NLR (*r* = 0.57, *p* = 0.003), neutrophil count (*r* = 0.526, *p* = 0.007), NIHSS (*r* = 0.607, *p* = 0.001), and infarct volume (*r* = 0.27, *p* = 0.051) (Fig. 1). Although the NLR increases as the NIHSS increases, this was not statistically significant (*p* value = 0.263). In contrast to the NIHSS, NLR was positively correlated with infarct volume (*r* = 0.35, *p* = 0.002).

Of the five genes in our panel, *ARG1* was the only gene that was significantly correlated with NIHSS (*r* = 0.708, *p* < 0.001). While the other four genes were not significantly correlated with NIHSS, several of the genes were significantly correlated with *ARG1* expression. Both *LY96* (*r* = 0.3, *p* = 0.048) and *MMP9* (*r* = 0.646, *p* < 0.001) expression were positively correlated with *ARG1* expression. *CCR7* expression was negatively correlated with *ARG1* (*r* = −0.381, *p* = 0.011).

After AIC model selection, there was a statistically significant association between *ARG1* and NLR (*p* = 0.019), adjusted for age, hypertension, and diabetes. There was also a statistically significant relationship between *ARG1* and NIHSS (*p* = 0.001), adjusting for age, sex, diabetes, prior stroke, and heart disease. After accounting for the adjusting variables, ARG1 explains 74.19 % of the variation in the reported NIHSS scale. Adjusting for prior stroke, there was also a moderate relationship between the NLR and NIHSS (*p* = 0.078). The median NLR was slightly higher in the moderate/severe stroke patients compared to mild but not statistically significant (mean NLR mild = 3.3, NLR moderate/severe = 4.5).

Prior to adjusting for regressors, there was not a statistically significant relationship between NLR and infarct volume (*r* = 0.347, *p* = 0.082). Although a regression model including all regressors suggested a statistically significant association between NLR and infarct volume (*p* = 0.022), the AIC selected model with only NLR (*p* = 0.006) and smoking status (*p* = 0.003) explained most of the variation in the infarct volume (adjusted R2 full model = 0.332 versus adjusted R2 AIC selected model = 0.458). After accounting for the smoking status of the patient, NLR explains a further 29.2 % of the variation in infarct volume. When adjusting for all regressors, there was no statistically significant relationship between *ARG1* and infarct volume (*p* = 0.627); this result held in the AIC selected model.

### Serum ARG1 Protein Activity, NLR, and Stroke Severity

**Fig. 2:**
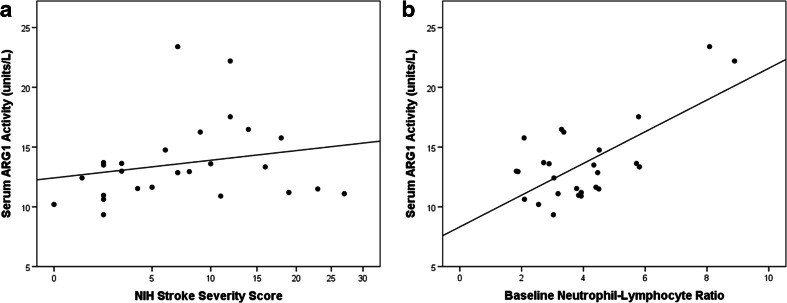
The relationship between serum ARG1 protein activity, NIHSS, and NLR. **a** Serum ARG1 protein activity is positively correlated with NIHSS (*r* = 0.31, *p* = 0.15). **b** Serum ARG1 protein activity is positively correlated with NLR (*r* = 0.52, *p* = 0.01)

Serum ARG1 protein activity is significantly correlated with whole blood *ARG1* messenger RNA (mRNA) expression (*r* = 0.502, *P* = 0.11). Similar to *ARG1* mRNA, there is a significant relationship between serum ARG1 protein activity and stroke severity, as measured by NIHSS. There is a moderately significant unadjusted relationship between serum ARG1 protein activity and NIHSS (*p* = 0.15) (Fig. 2). After adjusting for age, hypertension, smoking, heart disease, sex, hyperlipidemia, and diabetes, we observe that serum ARG1 activity has a statistically significant association with NIHSS (*p* = 0.027). After AIC model selection, there was a statistically significant association between serum ARG1 protein activity and NIHSS (*p* = 0.005), adjusted for heart disease and prior stroke.

There is a statistically significant positive correlation between serum ARG1 protein activity and NLR (*p* = 0.01) (Fig. 2). After adjusting for confounders, a moderately significant relationship between serum ARG1 activity and NLR remains (*p* = 0.062). After AIC model selection, adjusting for age, hypertension, and diabetes, there is a statistically significant relationship between serum ARG1 activity and NLR (*p* = 0.009).

### Gene Profile Validation in Stroke vs. Control

**Fig. 3:**
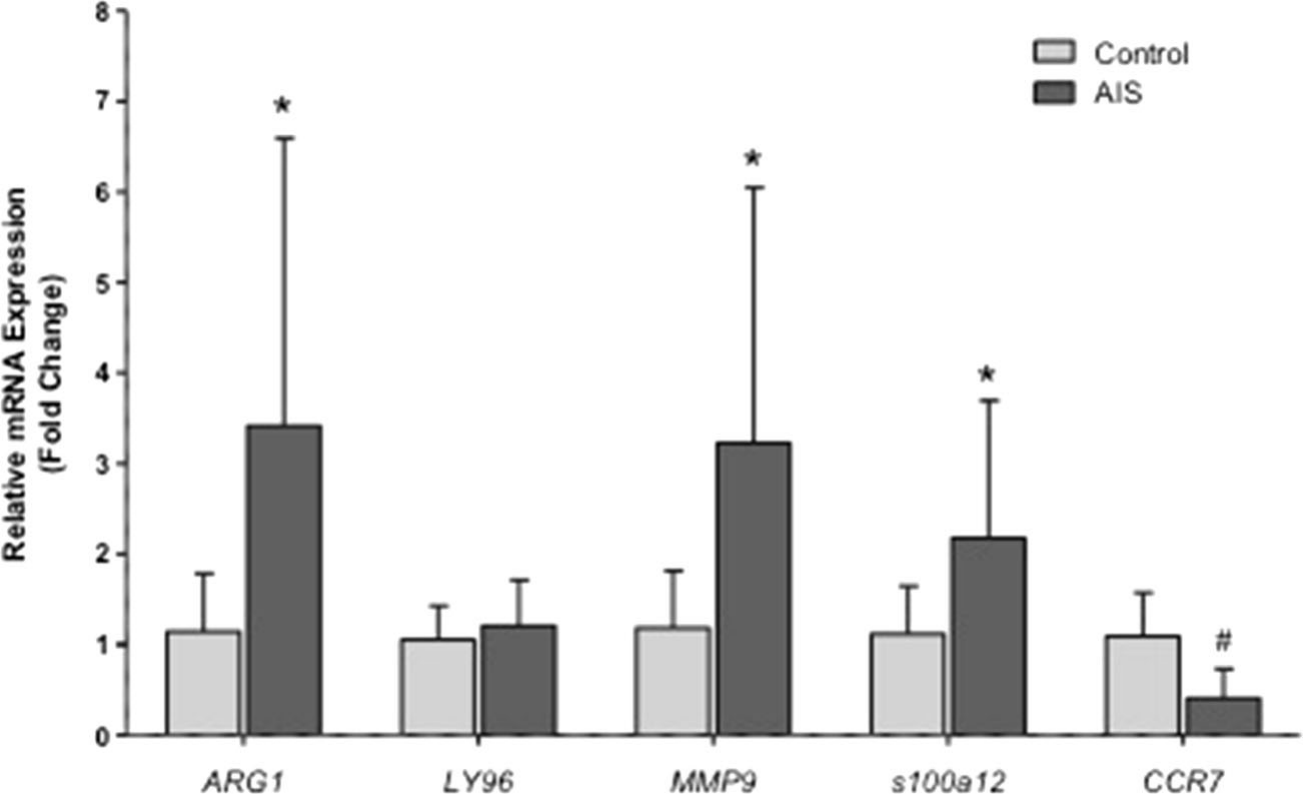
Relative expression of *ARG1*, *LY96*, *MMP9*, *s100a12*, and *CCR7* in AIS. *Significant increases in expression in AIS (*p* > 0.05). #Significant decreases in expression in AIS (*p* > 0.05)

Relative mRNA expression of *ARG1*, *MMP*, and *s100a12* was significantly increased in whole blood of stroke patients within 24 h of symptom onset compared to control (relative *ARG1* expression = 3.4 ± 3.2, *p* = 0.004; relative *MMP9* expression = 3.2 ± 2.8, *p* = 0.004; relative *s100a12* expression = 2.2 ± 1.5, *p* = 0.006). Relative mRNA expression of *CCR7* was significantly decreased in whole blood of stroke patients within 24 h of symptom onset compared to control (relative *CCR7* expression = 0.4 ± 0.32, *p* = 0.03). There was no significant difference in relative mRNA expression of *LY96* between stroke and control (Fig. 3). After AIC model selection, *ARG1* mRNA expression remained significantly higher in stroke compared to control (*p* = 0.001), adjusting for sex, diabetes, and prior stroke.

### Principal Component Analysis and Principal Component Regression

PCA with varimax rotation identified three principal components that explain 84.4 % of variation in the original patient gene dataset comprised of *ARG1*, *LY96*, *MMP9*, *s100a12*, and *CCR7* (Table 2). With *ARG1* and *MMP9* featuring large positive loadings and a large negative loading for *CCR7*, the first principal component (PC) can be considered a weighted contrast between *ARG1* and *MMP9* against *CCR7*; it explains 38.7 % of total variance (Table 2). The second PC is interpreted as a weighted average of *LY96* and *CCR7* and explains 24.4 % of the variation. The third PC is dominated by s100a12 and is interpreted as the contribution of s100a12 explaining 21.3 % of the variation.

**Table 2:** Loadings from the principal component analysis with varimax rotation using the five genes of interest: *ARG1*, *LY96*, *MMP9*, *s100a12*, and *CCR7*

	PC 1	PC 2	PC 3
*ARG1*	0.890	0.229	−0.153
*LY96*	0.307	0.875	−0.003
*MMP9*	0.852	0.016	0.102
*s100a12*	0.001	−0.064	0.991
*CCR7*	−0.570	0.630	−0.218
Explained variance (%)	38.7	24.4	21.3
Cumulative variance explained (%)	38.7	63.1	84.4

Principal component regression (PCR) was performed using the first three components and each outcome of interest: stroke versus control (non-stroke), NIHSS score, NLR, and infarct volume. Each PCR used all three of the derived components as regressors. The first PC was statistically significant in the PCR models with stroke versus control (*p* = 0.002), NIHSS (*p* < 0.001), and NLR (*p* = 0.005); it had moderate explanatory utility for infarct volume (*p* = 0.062). The second component was statistically significant in the PCR models with stroke versus control (*p* = 0.012) and infarct volume (*p* = 0.043). The third component was not statistically significant in any of the four PCR models.

## Discussion

The primary purpose of this study was to examine the relationship between ARG1, AIS severity, and immune status as measured by the NLR. We hypothesized that increased ARG1 expression is associated with greater AIS severity and immune dysfunction, as measured by an increased NLR.

This is the first report of a relationship between ARG1 and AIS severity. This study is the first to build upon existing literature which has shown that *ARG1* mRNA expression is increased in the peripheral blood, specifically by neutrophils, of AIS patients compared to control subjects; however, the association between ARG1 and increased severity had not yet been examined in the context of AIS. In support of our hypothesis, we observed a statistically significant relationship between increased ARG1, both at the gene and serum protein level, and increased AIS severity, as measured by both NIHSS and infarct volume.

In addition to the relationship between ARG1 expression and AIS severity, we also demonstrate a novel relationship between ARG1 expression and NLR. Here we report that increased ARG1 expression, at both the gene and serum protein level, is positively correlated with NLR and an increased neutrophil count. These associations were expected given the previous finding that *ARG1* mRNA expression is highest in the neutrophil fraction compared to other leukocyte populations [5]. Surprisingly, we report no relationship between ARG1 expression and lymphocyte count. It has been demonstrated previously that ARG1 protein released from neutrophils suppresses lymphocyte proliferation both in a human ex vivo model [10], as well as a murine AIS model [11]. Our results indicate that changes in ARG1 expression do not result in significant changes in lymphocyte count. This finding may be explained by the use of a single lymphocyte count, obtained within 24 h of AIS symptom onset. It may be possible that lymphocyte suppression measured via lymphocyte count may not be detectable until a later time point [4]. As ARG1 inhibits proliferation, there may not be an immediate change in cell count; rather, a prolonged suppression of lymphocyte proliferation would result in a reduced count over time.

Given the earlier findings by our laboratory, which demonstrate that an elevated NLR is associated with a poor outcome within 90 days of AIS, we hypothesized that the NLR may have a relationship with AIS severity within 24 h of symptom onset [8]. To address this hypothesis, we examined the relationship between NLR and AIS severity, measured by both NIHSS and infarct volume. While the NLR was higher in severe stroke (NIHSS >10) compared to mild (NIHSS <6), there was no statistically significant relationship between the NLR and NIHSS. In contrast, there was a statistically significant relationship between the NLR and infarct volume. These discrepant findings between two measures of AIS severity are not contradicting. The NIHSS provides a standardized assessment of neurologic deficit based on changes in cognitive and motor function, and in this study and previous, we have shown that NIHSS and infarct volume are not always correlated [17]. This is especially true in AIS patients with posterior circulation strokes, where very small infarcts can result in large neurological deficits and are reflected by higher NIHSS scores. The strong relationship between the NLR and infarct volume indicates that the magnitude of the immune response, as well as the factors that control that response, are a reflection of the amount of brain tissue compromised, rather than stroke-related symptom manifestation. In this study, the infarct volume was calculated based on imaging obtained near the time of blood sampling. Future studies will address how changes in both the NLR, as well as the factors that mediate the NLR, including ARG1, contribute to the evolution of brain infarct over time after AIS.

We validated the use of a gene profile comprised of five genes, *ARG1*, *LY96*, *MMP9*, *s100a12*, and *CCR7* to diagnose AIS. This validation was necessary given that previous validation studies utilized the reference gene β-actin; however, β-actin has been recently shown to be an unreliable reference gene due to variable expression levels in AIS [13]. We utilized a combination of two genes: *B2M* and *PPIB* that have been confirmed to be stably expressed in AIS. Compared to our previous study that had identified this panel of five genes associated with AIS, the relative expression of only one gene, *LY96*, was not increased in AIS patients in this separate cohort. The relative expression of the four remaining genes *ARG1*, *MMP9*, *s100a12*, and *CCR7* was comparable to previous studies.

In addition to validating the use of this gene profile in the diagnosis of AIS, we utilized PCA to examine the relationship between this panel of genes, AIS severity, and the NLR. We hypothesized that the pattern of expression among these genes would have better predictive value than studying single biomarkers alone. PCA represents an underutilized technique for studying the complex interactions giving rise to post-stroke immunosuppression. PC1 featured large positive loadings for *ARG1* and *MMP9* and a large negative loading score for *CCR7*, indicating an inverse relationship between *ARG1* and *MMP9* expression and *CCR7* expression. Further, PC1 is significantly correlated with AIS severity and NLR; and the relationships between PC1, AIS severity, and NLR are stronger than the relationships between *ARG1*, AIS severity, and NLR. These findings imply that recognizing patterns of biomarkers provides more clinical information than single biomarkers alone. While PCA is merely statistical, a potential physiological mechanism may be proposed based on these results. We postulate that the combination of increased *ARG1* expression and decreased *CCR7* expression results in an increased NLR. Because *CCR7* expression mediates lymphocyte migration to secondary lymphoid organs and lymphocyte activation, reduced *CCR7* expression may represent a mechanism of T lymphocyte suppression, mechanistically similar to the downregulation of CD3ζ chain [18]. Future studies, including human ex vivo and animal models of AIS, will be necessary to examine the functional relationship between *ARG1* and *CCR7*.

There are several limitations of this study that need to be addressed. The first limitation is the relatively modest sample size compared to other genomic biomarker studies. While the sample population in this study is limited, this sample size provides the required statistical power to address the primary aims of this study. The second limitation is the statistically significant age difference between the AIS and control groups, and the large age range within the AIS group. To consider the understanding that immune system function diverges with advancing age [19], we used linear regression analysis to statistically control for age and demonstrated that the relationships between ARG1, AIS severity, and NLR remain significant when accounting for age. This suggests that while the contribution of age to these relationships exists, there are other variables that are more significant contributors to these relationships. In addition to the large age range in the stroke subgroup, we did not acquire TOAST classification to determine AIS etiology.

This is the first report of a relationship between ARG1, infarct volume, and NLR in a human AIS model. This is also the first study to utilize PCA to model post-stroke immunosuppression. PCA is an underutilized technique that has clinical utility in the study of complex diseases, including AIS, and using PCA analysis we discovered novel relationships between multiple biomarkers that may play a physiological role in post-stroke immune suppression. The findings presented in this study have several implications for future clinical practice. ARG1 inhibition recently entered clinical trials for use in ischemia-reperfusion injury in patients with coronary artery disease [20]. Given the similar pathophysiological consequences in myocardial ischemia-reperfusion injury and AIS, it is plausible that ARG1 inhibition may represent a promising potential immune modulating treatment following AIS. Furthermore, because post-stroke immunosuppression persists for a period time in AIS recovery, ARG1 inhibition may have a beneficial effect on AIS severity and outcome even administered at a later time point than recanalization agents, such as rTPA.

## Conclusion

Our study suggests a novel relationship exists between ARG1, NLR, and stroke severity which may help guide future mechanistic studies of post-stroke immune suppression. The NLR is an underutilized clinically available biomarker to monitor the post-stroke immune response. Future studies will need to address the functional relationships between ARG1 and NLR post-stroke to test novel immune modulating therapeutic strategies for stroke patients.
